# Detection of Acute and Chronic *Toxoplasma gondii* Infection among Women with History of Abortion in the Southwest of Iran

**DOI:** 10.1155/2021/6693070

**Published:** 2021-11-02

**Authors:** Jasem Saki, Maryam Zamanpour, Mahin Najafian, Niloofar Mohammadpour, Masoud Foroutan

**Affiliations:** ^1^Infectious and Tropical Diseases Research Center, Health Research Institute, Ahvaz Jundishapur University of Medical Sciences, Ahvaz, Iran; ^2^Department of Medical Parasitology, School of Medicine, Ahvaz Jundishapur University of Medical Sciences, Ahvaz, Iran; ^3^Department of Obstetrics and Gynecology, School of Medicine, Ahvaz Jundishapor University of Medical Sciences, Ahvaz, Iran; ^4^Research Center for Environmental Contaminants (RCEC), Abadan University of Medical Sciences, Abadan, Iran

## Abstract

**Background:**

*Toxoplasma gondii* (*T. gondii*) is one of the most common intracellular protozoan parasites, which can infect humans and a wide range of mammals and birds. The current study is aimed at investigating the occurrence of *T. gondii* infection in women with a history of abortion in Khuzestan, Iran.

**Materials and Methods:**

A total of 480 women with an abortion history, as well as 200 pregnant women with a normal delivery, were examined in this study. The blood, placenta, and umbilical cord blood samples were assessed by the enzyme-linked immunosorbent assay (ELISA) and nested-polymerase chain reaction (PCR) assay.

**Results:**

Based on the results of ELISA assay, the prevalence of toxoplasmosis was 30.83% in women with a history of abortion (25.62% with *T. gondii* IgG and 5.20% with *T. gondii* IgM). According to the IgG avidity test, 60.16% of IgG-positive samples showed high avidity, while 27.64% showed low avidity. On the other hand, the prevalence of toxoplasmosis in women with a normal delivery was 23% (21.5% with *T. gondii* IgG and 1.5% with *T. gondii* IgM). According to the IgG avidity test, 81.39% of these women showed high avidity, while only 4.65% showed low avidity. Based on the nested-PCR method, *T. gondii* DNA was detected in 14.18% of blood samples, 4.69% of placental samples, and 1.34% of umbilical cord samples, collected from 148 seropositive women with a history of abortion. Besides, using this method, the parasite DNA was identified in 4.34% of blood samples, collected from 46 seropositive women with a normal delivery, but not in any of the umbilical cord or placenta samples.

**Conclusion:**

The present results showed that *T. gondii* infection contributes to abortion in Khuzestan Province, Iran. Therefore, it is essential to investigate toxoplasmosis in pregnant women, especially in those who are seronegative, using molecular and serological methods and inform them about their disease and the associated risks.

## 1. Introduction

*Toxoplasma gondii* (*T. gondii*) is one of the most common intracellular protozoan parasites, which can infect humans and a wide range of mammals and birds [1, 2]. Contamination with *T. gondii* primarily occurs due to the consumption of uncooked meat, infected with cysts, or the ingestion of oocysts through water, soil, and contaminated food. The incidence of this infection varies from one country to another, depending on the socioeconomic and health status [3–6]. Many cases of acquired toxoplasmosis are either asymptomatic or mildly symptomatic. However, in individuals with immunodeficiency, such as HIV patients and transplant recipients, it can be a life-threatening condition, causing eye and brain lesions. On the other hand, reactivation of a latent infection may cause severe complications with a poor prognosis [4, 7, 8]. Besides, infections during pregnancy can cause congenital toxoplasmosis in the fetus, resulting in spontaneous abortion, stillbirth, hydrocephalus, microcephaly, and neurological symptoms that can be detected in the uterus or at birth [9, 10].

According to previous studies, maternal infection in the first or second trimester of pregnancy can be associated with stillbirth rates of 5% and 2%, respectively [11, 12]. The prevalence of congenital toxoplasmosis varies from 0.1% to 0.3% per 1000 live births. The risk of maternal transmission to the fetus increases from 15% to 70% from a gestational age of 13 weeks to 36 weeks, respectively [13]. In a previous study conducted in New York, USA, 6% of pregnant women acquired toxoplasmosis during pregnancy, and 13% of their newborns had congenital toxoplasmosis. Overall, the prevalence of congenital toxoplasmosis was 7 per 10,000 live births [14].

The diagnosis of congenital toxoplasmosis in the uterus or after birth is essential in preventing and reducing severe complications in the fetus or newborn and improving the prognosis of infection [14, 15]. Generally, maternal serological examination for toxoplasmosis is crucial, especially in seroconverting mothers during pregnancy, to prevent fatal injuries through medical treatment or prophylaxis [16]. To identify infections during pregnancy more accurately, DNA-based molecular methods, which have higher sensitivity and specificity than serological methods, have been employed in recent years, and different targets of *T. gondii* genome have been investigated. The *B1* gene is one of the most valuable targets in identifying toxoplasmosis [17].

In some European countries, the monthly serological survey of sensitive pregnant women is a routine program, while in many countries, there is no information regarding congenital toxoplasmosis. Therefore, maternal and fetal lives can be threatened, especially in areas with a high prevalence of toxoplasmosis. In Khuzestan Province, with a population of approximately four million people, there is not enough information about congenital toxoplasmosis or the role of *T. gondii* in abortion. The present study is aimed at investigating anti-*T. gondii* antibodies and identifying parasite DNA in the maternal blood, placenta, and umbilical cord samples collected from two groups of women with a history of abortion and women with a normal delivery in hospitals, affiliated to Jundishapur University of Medical Sciences, Ahvaz, Iran, during 2012-2017.

## 2. Materials and Methods

**Figure 1:**
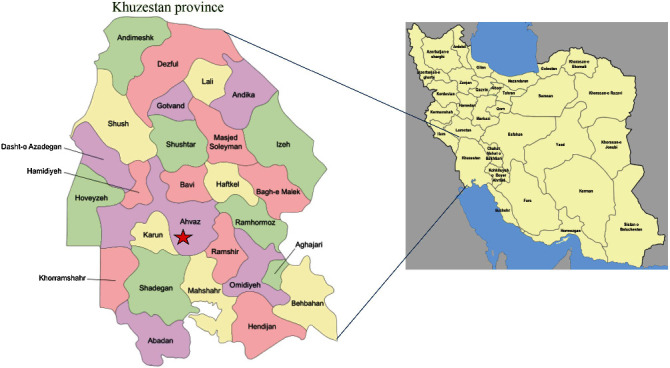
The map of Iran and Khuzestan Province in the southwest of Iran. The study region is shown with red asterisk.

Khuzestan Province is situated in the southwest of Iran (Figure 1). This study was carried out at the educational hospitals of Jundishapur University of Medical Sciences in Ahvaz, capital of Khuzestan Province, Iran. This study was approved by the Ethics Committee of Ahvaz Jundishapur University of Medical Sciences, Ahvaz, Iran. A written consent form was obtained from all women. Informed consent was also obtained from the participants' legal representatives.

From each patient (480 women with an abortion history and 200 with a normal delivery), peripheral blood samples, placenta, and umbilical cord samples (1-2 mL) were collected and transferred to the Parasitology Department of the School of Medicine. Next, serological enzyme-linked immunosorbent assay (ELISA) and molecular polymerase chain reaction (PCR) assays were carried out on the samples. A positive control was obtained from genomic DNA, extracted from the RH strain of *T. gondii*. The negative control for the PCR reaction was distilled water rather than DNA template, which is commonly used in PCR reactions.

### 2.1. ELISA Assay

A total of 480 peripheral blood samples were collected from women with a spontaneous abortion at <20 weeks of gestation, and 200 peripheral blood samples were collected from women with a normal delivery at a gestational age of 38-39 weeks. The participants in this study were in the age range of 14-53 years. The serological evidence of toxoplasmosis was investigated by detecting *T. gondii* IgM and IgG antibodies. The serum samples were separated and stored in aliquots at −20°C until further analysis. Next, they were tested for the presence of IgM and IgG antibodies, using an ELISA-based NovaLisa test kit. Moreover, a *Toxoplasma*-specific IgG avidity assay was performed using an avidity *T. gondii* IgG ELISA kit (NovaTec GmbH, Germany) to differentiate between acute and chronic infections. All samples were tested according to the manufacturer's instructions. The results were then read by an ELISA microplate reader (Bio-Rad Laboratories, Hercules, CA, USA) and compared with the calibrator and the controls.

### 2.2. DNA Isolation and PCR of *T. gondii B1* Gene

DNA was extracted from each blood sample, placenta, and umbilical cord sample (1-2 mL) (480 samples from women with an abortion history and 200 samples from women with a normal delivery), using a DNA extraction kit, according to the manufacturer's instructions (Bioneer, Korea). PCR was performed to amplify 194 bp segments of the *T. gondii B1* gene by nested PCR [18]. For the first and second rounds of PCR, 0.5 *μ*M primers, 200 *μ*M dNTPs, 1.5 mM MgCl_2_, 1.5 units of AmpliTaq gold DNA polymerase, and 4 *μ*L of DNA template were used in a total volume of 25 *μ*L. The PCR product was electrophoresed on 2% agarose gel. A 196 bp band was considered as the positive PCR control. Data were analyzed in SPSS version 13.5, using Chi-square and Fisher's exact tests.

This study was performed on 480 placenta, 480 umbilical cord, and 480 peripheral blood samples collected from women with an abortion, as well as 200 placenta, 200 umbilical cord, and 200 peripheral blood samples collected from women with a normal delivery, at university-affiliated hospitals in Ahvaz, Iran, during 2013-2017.

## 3. Results

### 3.1. Serological Results

Out of 480 blood samples collected from women with an abortion history by the ELISA method, 148 (30.83%) were positive, including 25 (5.20%) samples with IgM antibodies and 123 (25.62%) samples with IgG antibodies. According to the IgG avidity test, 74 out of 123 samples with IgG positivity (60.16%) showed high avidity; 34 (27.64%) samples showed low avidity, and 15 (12.19%) samples showed borderline avidity. In the control group, 46 out of 200 blood samples (23%) were positive in the ELISA assay. Three of these samples contained IgM antibodies (1.5%), while 43 (21.5%) contained IgG antibodies; only one sample included both IgG and IgM. The IgG avidity test showed that 35 (81.39%) and 2 (4.65%) samples had high and low avidity in this group, respectively (Table 1).

### 3.2. Molecular Test Results

**Figure 2:**
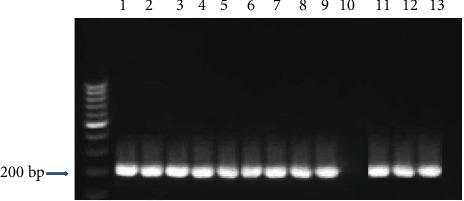
Amplification of a 194 bp band of *T. gondii* DNA in the blood, placenta, and umbilical cord samples. Lane M: molecular weight marker; Lane 1: positive control; Lanes 2-9 and 11-13: positive samples; Lane 10: negative control (2% agarose gel electrophoresis).

According to the results of nested-PCR assay in 148 seropositive women, *T. gondii* DNA was detected in 30 (20.27%) samples, which were collected from the group of women with an abortion history, including 21 (14.18%) blood samples, 7 (4.72%) placenta samples, and 2 (1.35%) umbilical cord samples. In this group, two women were positive in all samples (blood, placenta, and umbilical cord). Among other positive cases, 19 were only found in blood samples and five in only placental samples. On the other hand, in women with a normal delivery, only two women had serum-positive blood samples infected with *T. gondii*, while no infection was found in any of the placenta or umbilical cord samples (Figure 2 and Table 2).

## 4. Discussion

In this study, the prevalence of toxoplasmosis, based on serological methods, was estimated at 30.83% and 23% in women with a history of abortion and those with a normal delivery, respectively; the difference between the two groups was significant (*P* < 0.05). Based on the IgG avidity test, the rate of low avidity, which indicates a recent infection, was 27.64% versus 4.65% in women with a history of abortion versus women with a normal delivery, and there was a significant difference (*P* < 0.05). The significant difference observed in the IgM antibody levels of women with an abortion history (5.2%) and a normal delivery (1.5%) can indicate the involvement of *T. gondii* in abortion.

Various studies on toxoplasmosis in Khuzestan Province have reported the high prevalence of this parasitic infection. The seroprevalence of toxoplasmosis in women with a history of abortion was estimated at 24.6% in a previous study [19]. Moreover, IgG and IgM were detected in 29.3% and 7.9% of toxoplasmosis patients with renal failure, respectively [20]. Besides, soil contamination with *T. gondii* oocysts was reported in 9% of samples [21] and 16.5% of avian hosts [22]. Also, 38% of pregnant women (95% CI: 34-42%) were positive for IgG, while 4% (95% CI: 3-5%) were positive for IgM [23].

Toxoplasmosis, as a common global infection, is one of the causes of spontaneous abortion in humans and animals. During pregnancy, infection with *T. gondii* can cause congenital toxoplasmosis [24–26]. The rate of toxoplasmosis is relatively high among pregnant women in Iran. This rate has been estimated at 27% in Zahedan in the southeast of Iran [27], 60.6% in the north of Iran [28], 34.09% in Abadan (southwest of Iran) [29], 27.8% in Hormozgan Province [30], and 77.2% in Shiraz (south of Iran) [31]; overall, the rates were lower among high-school girls [32].

Moreover, in a study conducted in New York, USA, 0.6% of pregnant women acquired toxoplasmosis during pregnancy, and 13% of the newborns had congenital toxoplasmosis. Also, the rate of toxoplasmosis was 7 per 10,000 live births [14]. In another study in Colombia, 61 out of 15,333 cord blood samples contained IgM anti-*T. gondii* antibodies. Today, the prevalence of congenital toxoplasmosis is 39 per 10,000 live births [33]. A study on the seroprevalence of toxoplasmosis in pregnant women in Kosovo indicated that 1.2% of women had become infected with toxoplasmosis during pregnancy [34]. In the present study, the rate of *T. gondii* infection was 4.27% in the aborted placenta, according to the PCR method, which is 14.4% lower than the rate reported in Shiraz (south of Iran) [31]. It seems that the sample type, sampling method, and other parameters, such as climatic conditions and eating habits, can affect the prevalence of toxoplasmosis in different regions.

In 2017, Matin et al., in a comparison of placental PCR with maternal serology, showed that 53.5% of women with a history of abortion had anti-*T. gondii* antibodies; overall, 4% of these women had IgM, and 43% had IgG. A nested-PCR assay for identifying *T. gondii* in the placenta indicated that 10.5% of the samples were infected [35]. In the present study, infection with *T. gondii* was found in none of the placenta or umbilical cord samples of women with a normal delivery. The infection rate of the umbilical cord with *T. gondii* was 1.35% in women with an abortion history. Based on the results, the rate of umbilical cord contamination was lower than the placenta. These results are consistent with the results reported by Bessieres et al., detecting *T. gondii* in 60% of placenta samples and 43% of umbilical cord samples [36]. Some studies have also reported that detection of *T. gondii* in the placenta does not confirm congenital toxoplasmosis. In this regard, Sardarian et al., in 2018, tested ten placenta samples of women with a normal delivery and found that 6 (60%) samples were positive for *T. gondii* [37].

In conclusion, considering the significant differences in the prevalence of toxoplasmosis between the two groups of women with an abortion history and a normal delivery, *T. gondii* seems to contribute to the occurrence of abortion in Khuzestan Province. Therefore, it is essential to consider the risk of spontaneous abortion and stillbirth before and during pregnancy.

## Figures and Tables

**Table 1:** The serological results of toxoplasmosis in two groups of women with an abortion history and a normal delivery.

Groups	No.	IgM^+^ No. (%)	IgG^+^ No. (%)	IgG avidity^+^ No. (%)		Total^+^ No. (%)
				High avidity	Low avidity	
Women with an abortion history	480	25 (5.20%)	123 (25.62%)	74 (60.16%)	34 (27.64%)	148 (30.83%)
Women with a normal delivery	200	3 (1.5%)	43 (21.5%)	35 (81.39%)	2 (4.65%)	46 (23%)
Significance level		*P* < 0.05	*P* > 0.05	*P* < 0.05	*P* < 0.05	*P* < 0.05

**Table 2:** The molecular test results of toxoplasmosis in two groups of women with an abortion history and a normal delivery.

Groups	No. (seropositive)	Blood No. (%)	Placenta No. (%)	Umbilical cord No. (%)	Total No. (%)
Women with an abortion history	148	21 (14.18%)	7 (4.72%)	2 (1.35%)	30 (20.27%)
Women with a normal delivery	46	2 (4.34%)	0	0	2 (4.34%)
Significance level		*P* < 0.05	*P* < 0.05	*P* < 0.05	*P* < 0.05

## Data Availability

All relevant data are within the manuscript.

## Abbreviations

ELISA: Enzyme-linked immunosorbent assay
IgG: Immunoglobulin G
IgM: Immunoglobulin M
*T. gondii*: *Toxoplasma gondii*.
